# Disentangling interoceptive abilities in alexithymia

**DOI:** 10.1007/s00426-021-01538-x

**Published:** 2021-06-07

**Authors:** Cristina Scarpazza, Andrea Zangrossi, Yu-Chun Huang, Giuseppe Sartori, Sebastiano Massaro

**Affiliations:** 1 Department of General Psychology, University of Padua, Via Venezia 8, 35131 Padua, PD Italy; 2 Department of Neuroscience, University of Padua, Via Giustiniani, 5, 35128 Padua, Italy; 3 Padova Neuroscience Center (PCN), University of Padua, Via Giustiniani, 5, 35128 Padua, Italy; 4 The Organizational Neuroscience Laboratory, 27 Old Gloucester Street, London, WC1N 3AX UK; 5 Department of Psychology, University of Warwick, University Road, Coventry, CV4 7AL UK; 6 Surrey Business School, University of Surrey, Guildford, Rik Medlik Building (MS), Guildford, GU2 7XH UK

## Abstract

In recent years, research on interoceptive abilities (i.e., sensibility, accuracy, and awareness) and their associations with emotional experience has flourished. Yet interoceptive abilities in alexithymia—a personality trait characterized by a difficulty in the cognitive interpretation of emotional arousal, which impacts emotional experience—remain under-investigated, thereby limiting a full understanding of subjective emotional experience processing. Research has proposed two contrasting explanations thus far: in one model, the dimensions of interoceptive sensibility and accuracy in alexithymia would increase; in the other model, they would decrease. Surprisingly, the contribution of interoceptive awareness has been minimally researched. In this study (*N* = 182), the relationship between participants’ level of alexithymia and the three interoceptive dimensions was tested. Our results show that the higher the level of alexithymia is, the higher interoceptive accuracy and sensibility (*R*^2^ = 0.29 and *R*^2^ = 0.14); conversely, the higher the level of alexithymia is, the lower interoceptive awareness (*R*^2^ = 0.36). Moreover, an ROC analysis reveals that interoceptive awareness is the most accurate predictor of alexithymia, yielding over 92% accuracy. Collectively, these results support a coherent understanding of interoceptive abilities in alexithymia, whereby the dissociation of interoceptive accuracy and awareness may explain the underlying psycho-physiological mechanisms of alexithymia. A possible neurocognitive mechanism is discussed which suggests insurgence of psychosomatic disorders in alexithymia and related psychotherapeutic approaches.

**Supplementary Information:**

The online version contains supplementary material available at 10.1007/s00426-021-01538-x.

## Introduction

‘Interoception’ refers to the conscious perception and recognition of a wide range of physical internal states (Barrett & Simmons, 2015). The capacities to interocept, or interoceptive abilities, are central to the peripheral theories of emotions, which hold that emotions are perceived as central representations dependent on automatic bodily responses (Fehr & Stern, 1970). It follows that one’s ability to perceive more or less intensively their visceral responses, influences the strengths of the emotional experience (Critchley & Garfinkel, 2017; Garfinkel & Critchley, 2013): a high degree of interoception is supposed to reflect intense emotional experience and integration of bodily signals into emotional experience.

This understanding is particularly relevant for research on alexithymia, a personality trait which is characterized by a deficit in the cognitive interpretation of emotional arousal (Lopez-Munoz & Perez-Fernandez, 2019; Taylor et al., 2016), and therefore, impacts emotional experience. Originally defined as “absence of words for feelings” (Sifneos, 1973), this trait refers to a phenomenon characterized by difficulty in identifying one’s own and others’ feelings—in particular, negative emotions (Scarpazza et al., 2018; Sifneos, 1973; Starita et al., 2018; Taylor et al., 1991)—, difficulty in processing emotions (Nam et al., 2020), flattened affect and emotional unawareness (Sifneos, 1973; Taylor et al., 1991), low empathy (Alkan Hartwig et al., 2020; Moriguchi et al., 2007; Valdespino et al., 2017) and difficulties in social cognition (Di Tella et al., 2020; Lane et al., 2015; Moriguchi et al., 2006; Ospina et al., 2019; Scarpazza & Di Pellegrino, 2018). In other words, alexithymia would have prevented the Little Prince to express his emotions so vividly.

Research on alexithymia has blossomed in the past few years. For instance, the neurobiological underpinnings of alexithymia have been recently described as encompassing a complex neural network involving the insula, anterior cingulated cortex, amygdale, and prefrontal cortices, among other brain areas (Meza-Concha et al., 2017; van der Velde et al., 2013). Moreover, research has related alexithymia to multi-faceted difficulties in emotional processing, ranging from difficulties in recognizing emotions expressed by others (Grynberg et al., 2012; Scarpazza et al., 2014, 2015, 2018; Starita et al., 2018) to deficit in regulating emotional responses (Pollatos & Gramann, 2012; Swart et al., 2009). Furthermore, individuals with high alexithymia levels are defective in their ability to use emotions to guide their decision making (Scarpazza et al., 2017; Starita et al., 2019).

Thus, knowing more on the sub-clinical emotional processing impairment of individuals with high levels of alexithymia can offer a valuable opportunity to better evaluate the contribution of interoception to the conscious experience of emotions. Indeed, alexithymia is frequently acknowledged as “a marker of atypical interoception” (Murphy, et al., 2018; Murphy, et al., 2018): if individuals perceive and interpret their bodily sensations abnormally, they will also find challenging to identify and describe their feelings, as well as to regulate them when necessary.

While still embryonic, research has begun to focus on the cognitive underpinnings of alexithymia, with interoception emerging as the leading candidate (Murphy et al., 2018; Murphy et al., 2018; Nicholson et al., 2018; Scarpazza et al., 2015; Shah et al., 2016; Shah et al., 2016; Trevisan et al., 2019). Different theoretical models of interoception have been proposed in the literature thus far (Garfinkel & Critchley, 2013; Murphy et al., 2019), although, due to its complexity, it is a construct undergoing frequent refinement in both its conceptualization and operationalization (Trevisan et al., 2020). According with one popular model (Garfinkel & Critchley, 2013), interoception is a three-dimensional construct, in which each dimension reflects a different level of bodily signals elaboration. On a lower level, interoceptive sensibility (ISb) reflects a dispositional tendency to be internally focused (i.e., attention toward inner bodily signals); on a middle level, interoceptive accuracy (IAcc) refers to the objective accuracy in detecting internal bodily sensations; and on a higher level, interoceptive awareness (IAw) represents the meta-cognitive awareness of IAcc (Garfinkel & Critchley, 2013). Here, we build on this model of interoception, because the literature on alexithymia has primarily focused on IAcc and ISb thus far (see Online Supplementary Material) and left other interoceptive components advanced by more recent models (Murphy et al., 2019) still virtually unexplored in alexithymia. One of these components is for instance the dimension of attention—defined as the objectively measured attention to interoceptive signals measured with experience sampling methods (Murphy et al., 2019).

The involvement of IAcc and ISb in alexithymia has been explained by two competing hypotheses. One hypothesis suggests that individuals with high level of alexithymia—who lack of the ability to cognitively interpret bodily changes, with clear implication on their subjective emotional experience—are defective in interoceptive abilities (Bornemann & Singer, 2017; Brewer et al., 2016; Muir et al., 2017). According to this hypothesis, the difficulties manifested by alexithymic individuals to correctly identify their own emotions could be explained by a deficit in the accurate detection and identification of bodily changes, namely IAcc (Garfinkel & Critchley, 2013). This line of research also suggests a possible explanation for the higher occurrence of alexithymia in subjects presenting clinical disorders associated with poor interoception. For example, alexithymia is highly prevalent in individuals suffering from eating disorders, which are characterized by decreased interoceptive abilities related to reduced perception of hunger and satiety (Brewer et al., 2016). However, it is also worth to note that this hypothesis is difficult to reconcile with observations of increased prevalence of alexithymia in patients with psychosomatic disorders (Taylor, 2000; Taylor et al., 2016), whose attention is prevalently allocated on internal bodily signals.

A second hypothesis proposes that alexithymic individuals are characterized by heightened interoceptive abilities (Ernst et al., 2014; Longarzo et al., 2015; Scarpazza et al., 2015, 2017). This argument follows the “somatosensory amplification hypothesis” of alexithymia, which maintains that alexithymia is characterized by a perceived amplification of the normal visceral phenomena (Wise & Mann, 1994). This line of research appears to be prima facie inconsistent with previous studies reporting inter-dependence between subjective emotional experience and IAcc. At the same time, the precise contribution of IAcc and physiological responses to the conscious experience of emotion remains somewhat controversial (Lane & Schwartz, 1987; Scarpazza et al., 2015). The core proposition of this second hypothesis is that IAcc could be necessary but might not be sufficient for the conscious experience of emotion to arise. Indeed, emotional awareness can be graded in different levels, and accuracy in detecting bodily sensation are graded in the lower level (Lane & Schwartz, 1987). Although being able to detect their own visceral changes, alexithymic subjects may fail to link these signals to higher levels of emotional processing. Thus, this second line of research would not contradict, rather expand the current literature (Critchley & Garfinkel, 2017), suggesting the dissociation between interoceptive accuracy and emotional experience in alexithymia.

Altogether investigations of IAcc and ISb in alexithymia have provided contrasting results (see Online Supplementary Material for an overview of the literature). With such a mixed evidence supporting both perspectives, a recent meta-analysis has revealed a non-significant relationship between IAcc and alexithymia in a typically developing sample (Trevisan et al., 2019). Yet, to the best of our current knowledge (see also Online Supplementary Material for an overview of the literature), research regarding the association between the degree of interoceptive awareness and alexithymia is still missing (Scarpazza & Di Pellegrino, 2018). This is a rather surprising instance given that alexithymia represents a clear deficit in the cognitive interpretations of emotional arousal. Seeking to address this gap is the core aim of this study, which contributes to psychological theory by identifying the distinctive role that interoceptive abilities play in the (defective) processing of emotional experience, and to practice by putting forward clinical implications for addressing psychotherapeutic approaches for alexithymia. Thus, we hypothesize that individuals with a high level of alexithymia will be characterized by a decreased interoceptive awareness. In other words, they would lack self-confidence in their interpretation of bodily signals. This reasoning could thus explain why alexithymic individuals, despite being highly focused on bodily signals (Wise & Mann, 1994), are more prone to manifest disorders characterized by decreased interoception (Brewer et al., 2016).

## Materials and methods

### Participants

A priori power calculations using G*Power3 revealed that a sample of at least 161 participants is required to detect an association between interoception and alexithymia of *r* = 0.30 (Herbert et al., 2011), with a power of 0.99 using two-tailed tests. Participants were normal, healthy volunteers who replied to an online advertisement. Participants were included if they (i) declared they had never been diagnosed with any neurological or psychiatric disorder; (ii) were able to provide written informed consent; (iii) had a normal bodyweight; (iv) were proficient in both oral and written English.

A total of 193 participants, all with normal bodyweights (i.e., not obese), were recruited from the student and alumni population of our Universities, both in classes and through the SONA online recruitment system (https://www.sona-systems.com/). Participants were tested individually. Eleven participants were excluded because they reported feeling their pulse in the fingertip during the interoceptive accuracy task (see below). A total of 182 healthy participants (female = 95; age = 23.76; SD = 3.76) were retained for analysis. Data regarding age, gender, educational level and obesity (yes/no) were collected for control.

Ethical approval was received from the Ethical Review Board of the Warwick Business School (UK). The research was performed according to the principles of the Declaration of Helsinki.

### Variables and measures

#### Alexithymia

Participants completed the validated and widely used 20-item Toronto Alexithymia Scale (TAS-20) (Parker et al., 2003), which allowed us to evaluate their individual levels of alexithymia. The TAS-20 is a three-dimensional self-reported questionnaire, which measures three aspects of the alexithymia construct: difficulty in describing feelings (DDF), difficulty in identifying feelings (DIF), and externally oriented thinking (EOT). This self-report instrument has been demonstrated to have good psychometric properties: internal consistency Cronbach alfa = 0.81; test–retest reliability *r* = 0.86 (Bressi et al., 1996). In keeping with the current literature (Bagby et al., 2020), TAS-20 was used as a continuous variable in the regression models (see “Results” below). Moreover, for the ROC analyses (see “Results” below), following previous works that framed alexithymia as a binary status (Taylor et al., 2003), subjects were also classified as having high levels of alexithymia (HA) if their TAS-20 scores were ≥ 61 (*N* = 22), whereas they were classified as having low-level alexithymia (LA) if their TAS-20 scores were ≤ 60 (*N* = 160).

The Beck Depression Inventory-II (BDI-II) (Beck et al., 1961) was also administered, given that alexithymia has been strongly associated with depressive symptoms (Allen et al., 2011; Hintikka et al., 2001; Honkalampi et al., 2000; Li et al., 2015), and their co-occurrence might confound results. This instrument has demonstrated good psychometric properties (internal consistency Cronbach alfa = 0.91; test–retest reliability *r* = 0.93; (Beck et al., 1996, Beck et al., 1996).

#### Interoceptive sensibility

In keeping with previous research (Ernst et al., 2014; Palser et al., 2018; Pearson & Pfeifer, 2020; Scarpazza et al., 2015), the Body Perception Questionnaire (Porges, 1993) was adopted as a measure of Interoceptive Sensibility (ISb). This is a widely acknowledged self-reported questionnaire (see (Critchley, 2004; Garfinkel et al., 2015; Murphy et al., 2019) that measures one’s dispositional tendency to be internally focused and holds “high reliability and validity compared with other scales” (Ainley & Tsakiris, 2013). In the BPQ, participants were asked to indicate on a five-point Likert scale ranging from 1 (“never”) to 5 (“always”) their cognizance of bodily sensations and autonomic nervous system reactivity. The higher the score is, the stronger the participant’s subjective perception of bodily sensations and interoceptive sensibility.

#### Interoceptive accuracy

To allow a coherent comparison with the extant literature (Bekrater-Bodmann et al., 2020; Herbert et al., 2011; Nicholson et al., 2018; Scarpazza et al., 2015, 2017; Shah et al., 2016; Ueno et al., 2020), we used the heartbeat perception task—HBP task—(Schandry, 1981) to estimate Interoceptive Accuracy (IAcc) (Garfinkel & Critchley, 2013) (see “Discussion” below for benefits and limitations of this task).

During this task, participants were asked to count their heartbeats silently by focusing on their heart activity, while actual heartbeat signals were simultaneously acquired using a wireless finger pulse oximeter (DigiO2 International Co.) (Shah et al., 2016). Participants were not allowed to take their pulse or attempt any other physical manipulation that could facilitate the heartbeat count. Furthermore, participants were instructed not to guess if they could not feel their heartbeat. This task was repeated three times to form three trials, using time-windows of 30, 45, and 60 s, presented in randomized order. IAcc was calculated by taking the mean score across the three heartbeat perception intervals according to the following transformation: 1/3 ∑ (1−(|recorded heartbeats – counted heartbeats|)/recorded heartbeats) (Schandry, 1981; Schuette et al., 2020). The resulting score, also called *heartbeat perception index* or *interoceptive accuracy index*, was calculated following previous works that adopted this task (Herbert et al., 2011; Schandry, 1981; Schuette et al., 2020; Shah et al., 2016); the score varies between 0 and 1, where 1 indicates the highest accuracy (Schandry, 1981; Schuette et al., 2020). After completion, participants were debriefed on the eventual adoption of exteroceptive strategies: they were explicitly asked whether they were able to feel their pulse in the fingertip where the pulse oximeter was clamped, or whether they used other strategies in performing the task (e.g., counting the time). Eleven participants disclosed that they felt their pulse in the fingertip and were discarded from the analysis; no participant reported counting the time or using other strategies.

#### Interoceptive awareness

In keeping with existing research (Bekrater-Bodmann et al., 2020; Garfinkel et al., 2015; Khalsa et al., 2020), at the end of each heartbeat perception task trial, participants were asked to rate how confident they were about their performance at each IAcc trial. This rating was performed using a paper/pencil to mark a Likert Scale ranging from 0 (“Total guess/I believe that my performance on the task was extremely poor”) to 10 (“Complete confidence/ I believe that my performance on the task was very good”).

Since interoceptive awareness (IAw)is defined as the way in which confidence in the task performance reflects IAcc (Garfinkel et al., 2015), the ratio between IAcc (reported on a 0–10 scale to match the confidence values) and confidence, as measured above, was computed to index an estimate of participant-specific IAw. A resulting value of IAw = 1 indicates a perfect correspondence between IAcc and confidence (e.g., IAcc = 5; confidence = 5: IAw = 5/5 = 1); values of IAw > 1 indicate participants with higher IAcc than confidence (e.g., IAcc = 5; confidence = 3: IAw = 5/3 = 1.66), while values of IAw < 1 indicate participants with lower IAcc than confidence (e.g., IAcc = 3; confidence = 5; IAw = 3/6 = 0.6). Thus, this index allows us to say that both individuals with low IAcc and low confidence (e.g., IAcc = 2; confidence = 2: IAw = 1; indicating that a subject is aware that their abilities to discriminate bodily sensation are not good) and individuals with high IAcc and high confidence (e.g., IAcc = 9; confidence = 9: IAw = 1; indicating that a subject is aware that their abilities to discriminate bodily sensation are not good) have high awareness of their bodily sensations. Here, the variable IAw was used as a continuous variable (i.e., participants were not grouped depending on their IAw score).

### Statistical analyses

Three linear regression models were built, using the total TAS-20 score as a continuous dependent variable. The normality of the TAS-20 score was assessed by means of the Shapiro–Wilk Test (*W* = 0.99, *p* = 0.11). Each model included one only among ISb, IAcc, and IAw as the predictor of interest, while controlling for age, gender, and depressive symptoms. This was made necessary by the fact that IAw was calculated as a ratio between IAcc and confidence, and thus IAw and IAcc are not independent. As a consequence, including both IAcc and IAw within the same model would have led to collinearity issues. For each individual model, we tested the assumption of normality of the residuals (Shapiro–Wilk test *p* = 0.09, 0.19, and 0.13, respectively) and checked for collinearity across predictors by means of variance inflation factors (VIF). VIF resulted, in every case, in a score below 10, indicating the absence of multicollinearity (Bowermann & O'Connel, 1990; Myers, 1990). The presence of influential outliers was checked by means of the Cook’s distance (Di), which resulted, in every case, in a score lower than 1, allowing us to rule out their presence (Cook & Weisberg, 1982).

The regression models were compared by assessing both their absolute and relative goodness-of-fit. The former was measured by the amount of explained variance in the model (*R*^2^), while the latter was evaluated by means of both the Akaike Information Criterion (Akaike, 1987) and the Bayesian Information Criterion (Schwarz, 1978). Specifically, AIC and BIC evaluate a model’s parsimony (i.e., the balance between the inclusion of more predictors and the related increase in model fit). Simply put, lower values of these measures indicate better fitting models. We also employed the Bayes Factor (BF) as a measure of relative likelihood (Kass & Raftery, 1995; Lavine & Schervish, 1999) to directly compare one model against the others.

Moreover, we tested the ability of each model to predict the TAS-20 score in a Leave-One-Out Cross-Validation (LOOCV) design. That is, one observation was left out and each model was built using *N-1* observations; the left-out value was then predicted. This procedure was repeated *N* = 182 times, until each and every observation was left out and predicted once. The prediction accuracy was assessed through correlation between the actual and predicted TAS-20 values.

In addition, we compared ISb, IAcc, and IAw to identify individuals with high alexithymia levels by means of a receiver-operating characteristic (ROC) analysis, now using alexithymia as a binary variable. That is, this analysis was designed to determine the strength to which a variable can predict a binary state (i.e., high vs low levels of alexithymia based on the TAS-20 cutoff reported above) while testing all possible thresholds or cutoff values. Thus, the number of correctly classified elements and errors (i.e., subjects classified as belonging to their actual class and those wrongly classified, respectively) was computed for all predictor values as a potential detection threshold. In practice, an ROC analysis results in a curve showing the relation between sensitivity and specificity for each tested threshold, allowing the best cutoff value to be identified. In this way, it is possible to compare the accuracy of the three interoceptive dimensions in the correct identification of individuals with a high level of alexithymia. The best detection threshold, which as we shall discuss is IAw, has the highest accuracy, maximizing both sensitivity and specificity. All analyses were performed in R (R Core Team, 2013).

## Results

The mean TAS-20 was 46.3 ± 11.29 (DDF: 13.59 ± 4.5; DIF: 16.36 ± 5.35; EOT: 16.37 ± 4.37). Twenty-two participants reported high levels of alexithymia (12.08% of the sample), which is a value fully aligned to the general population in which alexithymia has an average prevalence of 10% (Kokkonen et al., 2001; Muir et al., 2017; Taylor et al., 1991). Thus, we were confident that our sample could be considered representative of the general healthy population. Moreover, the mean ISb was 231.8 ± 46, and the mean IAcc index was 0.58 ± 0.18, suggesting moderate accuracy in the identification of bodily sensations; the mean IAw index was 1.11 ± 0.60, suggesting good awareness of bodily sensations.

### Regressions using interoceptive dimensions as predictors

**Fig. 1:**
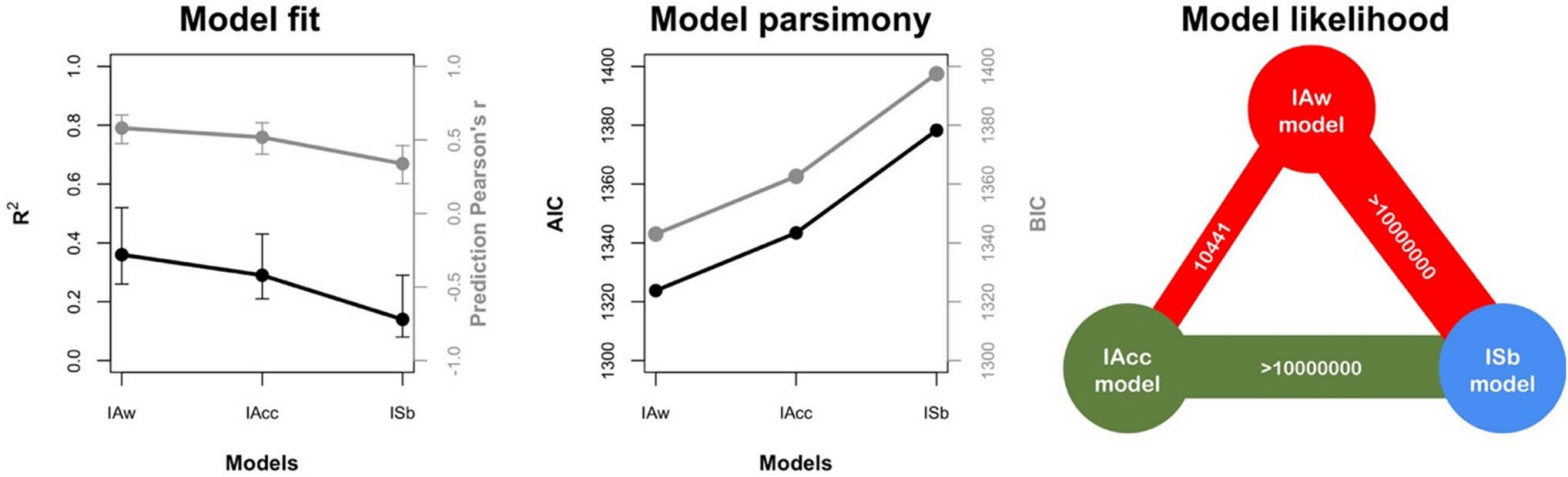
Models’ comparison. On the left panel: the percentage of explained variance of each model (*R*^2^) and the correlation between actual and model-predicted TAS-20 scores (Pearson’s *r*) are reported. On the central panel: the models’ parsimony is evaluated by means of the Akaike Information Criterion (AIC) and the Bayesian Information Criterion (BIC), with lower values indicating better models. On the right panel: comparisons among all models in terms of likelihood, by means of the Bayes Factor (BF). Lines’ width indicate BF magnitude while the colors indicate the best model for each comparison (red = IAw model, green = IAcc model, blue = ISb model) (color figure online)

All the three models including one interoceptive dimension as predictor and TAS-20 as dependent variable showed a significant effect of the interoceptive dimension considered (Table 1). However, using the model comparison procedure described above, the model including IAw as predictor of interest was identified as the best one to explain the TAS-20 total score (Fig. 1). That is, the IAw model showed the highest amount of explained variance (R^2^; Fig. 1—left, black line), the highest likelihood (BF; Fig. 1—right), and the best prediction performance (Pearson’s *r* between actual and predicted TAS-20 values created using the LOOCV design; Fig. 1—left, grey line), relative to the other models. Concurrently, this model minimized both AIC and BIC (Fig. 1—center), thus providing the optimal trade-off between model complexity and data explanatory power.

**Table 1:** Results of the linear regression models

	Models	Model statistics			Predictors	*β*	SE	*t*	*p*
		*F* (*df*)	*p*	Adjusted-R^2^					
Predictor of interest	IAw Model	26.77 (4177)	< 0.001	0.36	Age	− 0.28	0.18	− 1.55	0.12
					Gender	0.15	1.37	0.11	0.91
					BDI	0.01	0.12	0.09	0.93
					IAw	11.42	1.11	10.25	< 0.001
	IAcc Model	19.51 (4177)	< 0.001	0.29	Age	− 0.26	0.19	− 1.37	0.17
					Gender	0.40	1.44	0.28	0.78
					BDI	− 0.04	0.13	− 0.32	0.75
					IAcc	34.38	3.94	8.73	< 0.001
	ISb Model	8.39 (4177)	< 0.001	0.14	Age	− 0.29	0.21	− 1.40	0.16
					Gender	− 0.36	1.59	− 0.23	0.82
					BDI	− 0.13	0.14	− 0.87	0.39
					ISb	0.09	0.02	5.67	< 0.001

**Fig. 2:**
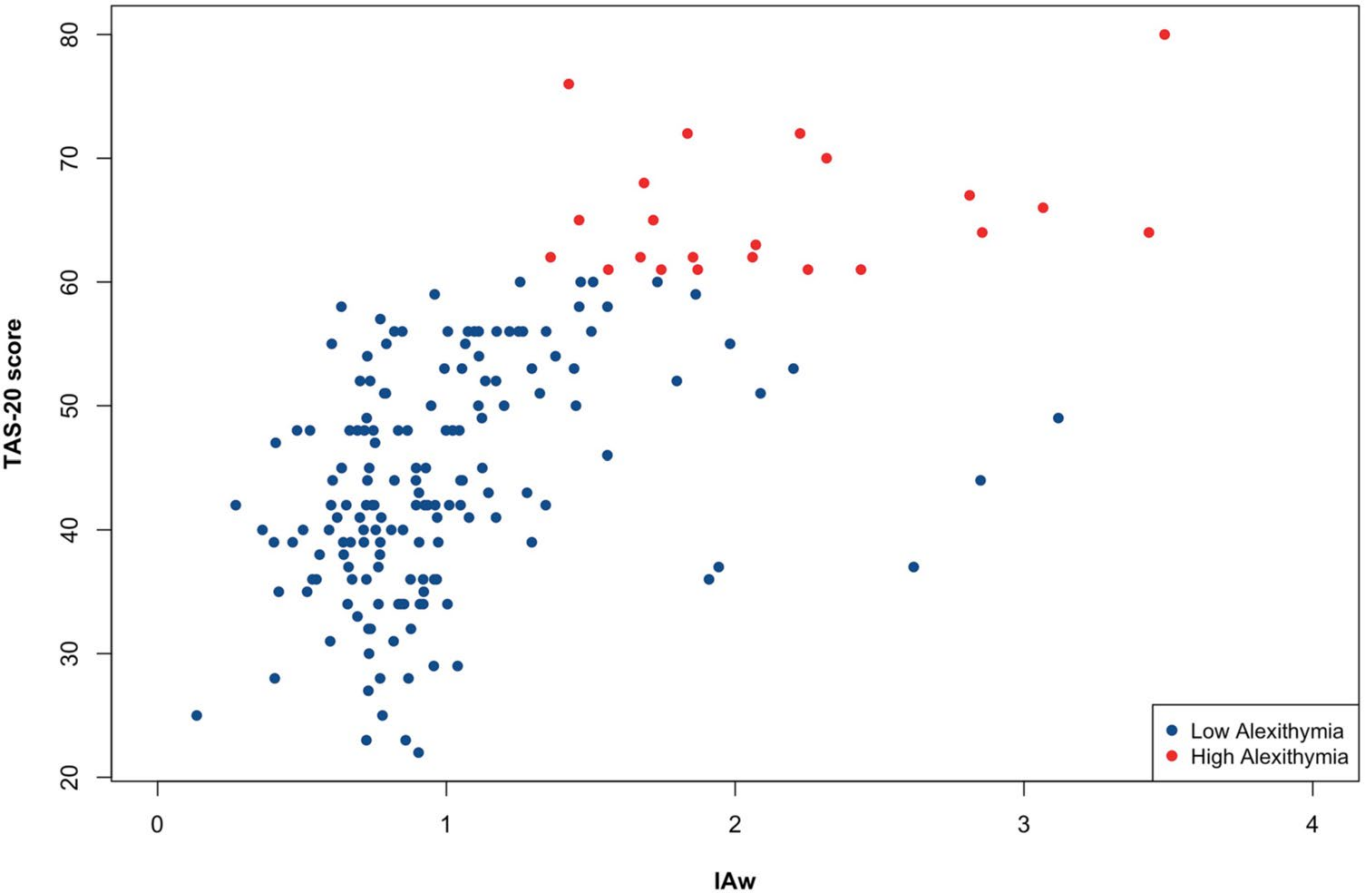
Relationship between interoceptive awareness and alexithymia (TAS-20 = 20-items Toronto Alexithymia Scale total score)

The regression model using TAS-20 total score as a continuous dependent variable and IAw as predictor of interest was statistically significant [*F*(4, 177) = 26.77; *p* < 0.001; adjusted R-squared = 0.36] and revealed a significant effect of IAw [*t*(177) = 10.25; *p* < 0.001; 95% C.I. (9.2, 13.6)], but no significant effects of age, gender, or depressive symptoms, as shown in Table 1. Intriguingly, the analysis revealed that the higher the IAw index is, the higher the alexithymia level. Since a high IAw index identifies individuals with higher IAcc than confidence, these results indicate that the higher the alexithymia level is, the lower the interoceptive awareness. These results are shown in Fig. 2.

The regression models using TAS-20 total score as dependent variable and IAcc and ISb as predictors of interest were also statistically significant (see Table 1), showing a significant effect of IAcc and ISb [*t*(177) = 8.73 and *t*(177) = 5.67 for IAcc and ISb, respectively], but no significant effect of age, gender or depressive symptoms. The analyses revealed that the higher the IAcc and ISb indices are, the higher the level of alexithymia.

### Interoception dimensions and alexithymia prediction

**Fig. 3:**
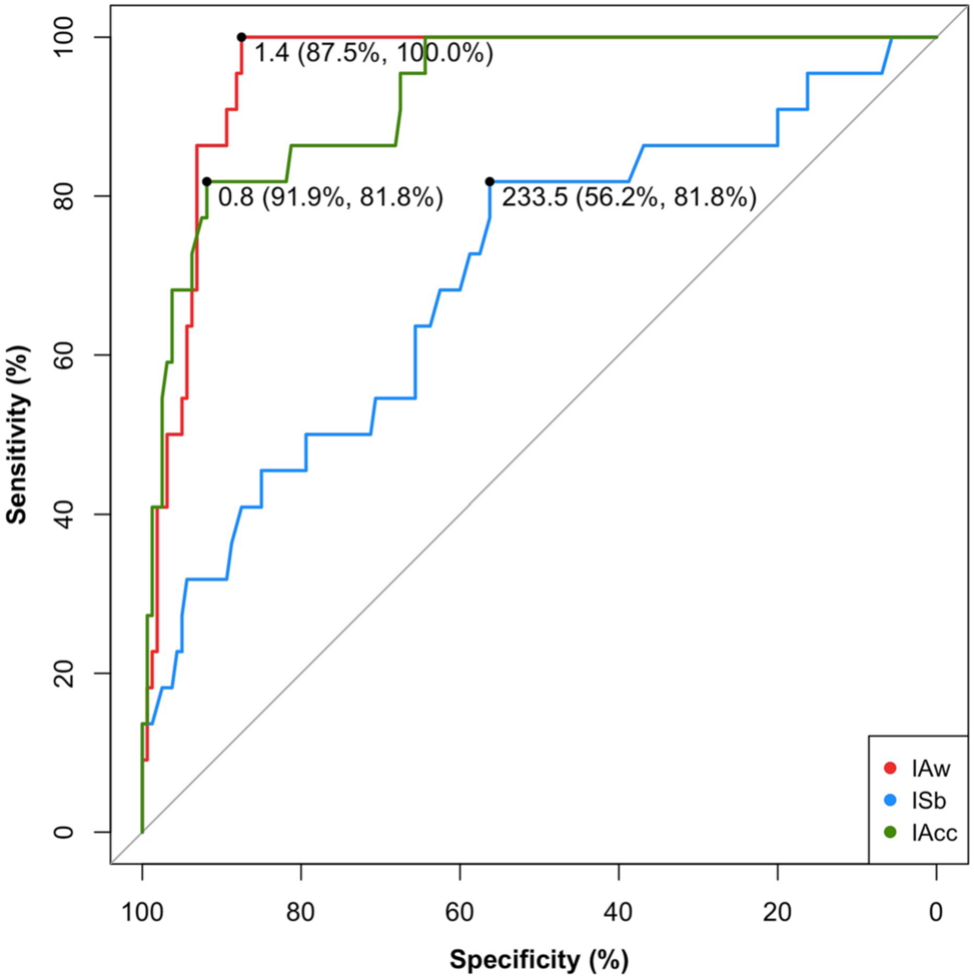
ROC curve showing sensitivity and specificity of the models testing the relative contribution of the three interoceptive dimensions for alexithymia prediction. For each curve, a black dot indicates the best cutoff value and the corresponding specificity and sensitivity (color figure online)

The ROC analysis showed that the three dimensions of interoception offer different levels of accuracy in the prediction of alexithymia as a binary state, as summarized in Fig. 3. IAw is the interoceptive dimension that most accurately predicted alexithymia in our sample. Among all the tested thresholds, an IAw cutoff value of 1.4 shows the highest accuracy at 93.8% [sensitivity = 100%; specificity = 87.5%; AUC = 95.4%, 95% CI (92.5–98.2%)]. In other words, IAw correctly classified all the 22 individuals with high alexithymia level, and 140 out of 160 individuals without high alexithymia level.

IAcc also proved to be a good predictor: Among all the tested thresholds, an IAcc cutoff value of 0.8 showed the highest accuracy, at 86.9% [sensitivity = 81.8%; specificity = 91.9%; AUC = 92.5%, 95% C.I. (87.4–97.8%)]. That is, IAcc correctly classified 18 out of 22 individuals with high alexithymia level, and 147 out of 160 individuals without high alexithymia level.

ISb proved to be a less accurate predictor of alexithymia than the other interoceptive dimensions. Among all the tested thresholds, an ISb cutoff value of 233.5 showed the highest accuracy, at 69% [sensitivity = 81.8%; specificity = 56.2%; AUC = 70.1%, 95% C.I. (57.6–82.7%)]. In other words, ISb correctly classified 18 out of 22 individuals with high alexithymia level, and 90 out of 160 individuals without high alexithymia level.

## Discussion

Alexithymia is generally considered to be “a marker of atypical interoception” (Murphy et al., 2018; Murphy et al., 2018). Yet, research on interoceptive abilities in alexithymia is still in its infancy. Specifically, the association/dissociation of the three interoceptive dimensions in alexithymia has received little attention thus far, with the dimension of interoceptive awareness remaining under-investigated (see Online Supplementary Material for an overview).

Here, we examined the link between these dimensions and the construct of alexithymia. The results we obtained using the heartbeat perception task reveal that, among all interoceptive dimensions, IAw can most reliably predict alexithymia. This is because the higher the TAS-20 total score, the lower the IAw. Moreover, we show that interoceptive abilities are dissociated in alexithymia: IAcc and ISb increased for higher alexithymia levels, while IAw decreased for higher levels of alexithymia. Finally, IAw and IAcc can accurately identify alexithymic participants within our sample.

The functional meaning of these results indicates that individuals with a high level of alexithymia, despite being more focused on their bodily sensations (in accordance with the original “somatosensory amplification hypothesis”; (Wise & Mann, 1994) and being more capable of detecting their own bodily signals (in accordance with the second line of research) compared with individuals with low alexithymia, may lack self-confidence in their bodily signals, reporting to not feel their judgement of their own bodily sensations is trustworthy (). This would then result in an overall deficit in interoception. This finding aligns with recent theorizations proposing that alexithymic individuals do not present difficulties in perceiving or reporting internal body sensations, but rather have difficulty in interpreting their bodily sensations (Fournier et al., 2019; Zamariola, et al., 2018).

Collectively, this study’s contribution is threefold. First, our results indicate a possible integration of the two contrasting hypotheses of interoception and alexithymia. Second, they suggest a promising neurocognitive mechanism for higher risk of psychosomatic disorders in alexithymia. Third, this work opens the way for promising psychological interventions to modulate difficulties experienced by alexithymic individuals.

As regards our first contribution, this work’s results support both existing models of interoceptive abilities in alexithymia. Since interoceptive accuracy may be a necessary, yet not a sufficient feature for the conscious experience of emotions (Critchley & Harrison, 2013), a dissociation between interoceptive accuracy and awareness may represent the core factor underlying alexithymic deficits. Indeed, emotional awareness can be graded in different levels, and objective accuracy in detecting bodily sensation is graded in the lower level (Lane & Schwartz, 1987). While individuals with high level of alexithymia could be characterized by heightened perception of bodily sensation, they may also put a ‘gridlock’ on a lower level of emotional experience, without being able to link their visceral signals to higher levels of emotional processing. In this way, emotion-evoking situations would be perceived only at the physical level and remain void of any emotional implication (Scarpazza et al., 2015). This issue would prevent the formal and symbolic representation of emotions as a tool for effective emotional regulation (Schuette et al., 2020). In other words, our results provide support for the idea that individuals with high levels of alexithymia believe that they are not able to feel the internal sensations, manifesting a dissociation between their interoceptive accuracy and awareness. This explanation would thus offer a coherent lens to understand the currently fragmented results present in the literature. Yet, we shall also note that impoverished interoceptive awareness would support the first theoretical model, which claims a deficit of interoception in alexithymia. Congruently, functional hypoactivation in the anterior part of insula (Bird et al., 2010; Hogeveen et al., 2016; Lemche et al., 2013) and hyperactivation of the posterior part (Goerlich-Dobre et al., 2014; Wiebking & Northoff, 2015) have been observed in individuals with high levels of alexithymia. In addition, grey matter volume increases in the posterior insula (Goerlich-Dobre et al., 2014) and decreases in the anterior insula (Borsci et al., 2009; Ihme et al., 2013) have been observed in individuals with a high alexithymia level, as compared to individuals with low alexithymia levels. Research has also shown that individuals with structural acquired damage to the anterior insula manifest acquired alexithymia—i.e., alexithymia emerging as a consequence of brain damage (Hogeveen et al., 2016). Indeed, the insula, which is well documented to be one of the brain regions most associated with interoception (Allen, 2020; Critchley, 2004; Critchley et al., 2004), is characterized by an anterior–posterior gradient: the posterior insula is mainly responsible for allowing the perception of visceral/bodily signals, while the anterior insula integrates the bodily sensations with subjective feelings and awareness (Craig, 2011).

The second contribution of our work allows us to propose a potential neurocognitive mechanism to explain the increased risk of psychosomatic disorders in alexithymia. Due to their higher IAcc, individuals with high levels of alexithymia would be more accurate in the perception of emotion-related physiological reactions (Wise & Mann, 1994). However, due to their low IAw abilities, they would be unable to correctly interpret their bodily/visceral changes as emotions, and instead misinterpret them as a bodily symptoms (Scarpazza et al., 2015). This process could lead to somatization. This argument is in line with other evidence present in the literature. For example, it resonates with observations that alexithymic individuals delay seeking medical treatment (Carta et al., 2013). This behavior, which was previously explained by a deficit of ISb or IAcc (Brewer et al., 2016), is likely to be due to low interoceptive awareness: Individuals with a high level of alexithymia are likely to mistrust their physiological states and not to be confident of their ability to detect their bodily states. Most of all, this conceptualization reflects the original definition of alexithymia as a deficit in the cognitive interpretation of emotional arousal (Taylor, 2000) and with the first version of the TAS scale, in which a factor called “difficulty in distinguishing between feeling and bodily sensations that accompanied emotional arousal” was originally included ().

Finally, the current study provides preliminary insights useful to both the development and implementation of psychotherapeutic interventions in alexithymia which is unresponsive to classical psychotherapies (Taylor, 2000). Intervention specifically designed to improve interoceptive awareness and to enhance the ability to correctly interpret emotion-related bodily changes may benefit alexithymic individuals’ social life and mental health (Duquette, 2020; Shalev, 2019). Since alexithymic individuals experience strong visceral responses (Wise & Mann, 1994) without being able to cognitively interpret them, these individuals are thought to be “at the mercy” of their bodily sensations, which might prevent them from effectively selecting suitable strategies for emotion regulation. Not surprisingly, the sole psychotherapies showing marginal effectiveness in alexithymia are those emphasizing the necessity to enhance emotional awareness (Taylor, 2000). Our work suggests that a possible intervention can be placed on interoceptive awareness, given its pivotal role in a correct reappraisal of emotional responses (Duquette, 2020; Fustos et al., 2013; Shalev, 2019). While we call for future research to replicate our results using additional interoceptive measures (see “Limitations” below), the potential future implications of our study are widespread, given that alexithymia is not only a critical component in some psychiatric disorders (De Panfilis et al., 2015), but it is also a mediating factor of mental health problems in stressful environmental situation, such as the recent COVID-19 pandemic (Tang et al., 2020) and it is closely associated with aggressive behavior (Li et al., 2020). It is thus clear that identifying a way to effectively modulate alexithymia and the association between alexithymia and interoceptive dimensions appears to be pivotal to prevent both mental health problems and the overt expression of aggressive behaviors in vulnerable individuals.

### Limitations and future research avenues

This study should also be seen in the light of its limitations, as well as opportunities for future research. The main limitation of this study is inevitably linked to the rapid evolution of the definition of interoception, a complex and multi-faceted construct that is undergoing continuous refinement in conceptualization and operationalization. This issue concerns, in particular, the heartbeat perception task—HBP task—that we applied in the current paper to measure interoceptive accuracy by following the previous literature on alexithymia and interoception (Bekrater-Bodmann et al., 2020; Herbert et al., 2011; Nicholson et al., 2018; Scarpazza et al., , 2015, 2017; Shah et al., 2016; Ueno et al., 2020). Despite its extensive and current use, this task has recently been questioned in terms of its capacity to effectively capture IAcc (Desmedt et al., 2018; Ring et al., 2015; Zamariola, et al., 2018). Some of the reasons why the task has been criticized as a measure of IAcc are well summarized in (Murphy, et al., 2018). First, heartbeats may be perceived via (exteroceptive) touch receptors due to the vibration of the chest wall (Brener & Ring, 2016; Desmedt et al., 2018; Khalsa et al., 2009); the extent to which the heartbeat may be perceived exteroceptively depends on factors such as the percentage of body fat and systolic body pressure. Thus, future research might consider adding further control measures possibly impacting task performance, such as body mass index, systolic blood pressure, heart rate variability (Castaldo et al., 2017; Massaro & Pecchia, 2019) among others. In this work, we did not collect such variables; however, at the completion of the task, we probed participants on whether or not they felt the pulse, as a way to control for possible exteroceptive strategies, and we excluded participants who did. Given that the inclusion of these variables in previous research showed modest influence on the effect size of the relationship between alexithymia and HBP task performance (Murphy, et al., 2018), we can reasonably suppose that our results were not greatly affected by the absence of such control variables.

Second, the knowledge of one’s own, or the average person’s heart rate may impact the results obtained using the HBP task (Ring et al., 2015). A growing body of research demonstrates that manipulating participants’ beliefs about one’s own resting heart rate may alter heartbeat counting estimates in the HBP (Ring et al., 2015). Similarly, accurate knowledge of average heart rate correlates with improved performance on the HBP task (Murphy et al., 2018). Although, in this paper, we did not probe the participants about their knowledge of their heart rate, the results were corrected for depressive symptoms and gender, with the latter variable having a stronger impact on the task performance than mere knowledge of resting heart rate (Murphy et al., 2018).

Third, the HBP task results may be affected depending on whether participants are encouraged to guess if they cannot feel their heartbeat (Murphy, et al., 2018). If participants are instructed to guess (or if they do so regardless of the instruction not to), then a sensible strategy is to estimate the duration of the interval over which one is required to count one’s heartbeats and/or to count seconds instead of heartbeat to arrive at an estimate of the number of heartbeats. Here, after task completion, we asked participants whether they counted seconds instead of heart beats and no participant reported counting the time or using other strategies.

Additional issues associated with the HBT that might have affected our study’s results are described in (Zamariola, et al., 2018) They can be summarized as follows: (i) HBT does not distinguish between over and underestimation of heartbeats; (ii) the correlation between actual and reported heart rate is low; (iii) the IAcc scores vary across the time intervals used in the task. We did not consider these issues in the current study, which might thus limit the robustness of our findings. However, it is worth noting that, Murphy et al., (2018) recently showed consistency among results obtained using multidimensional interoceptive tasks, including the heartbeat perception task, thus giving us confidence in its inferential capabilities for the purposes of this study.

It is also worth noting that there is increasing evidence providing support for the notion that heartbeat perception might be a good index for interoception research. Several functional neuro-imaging studies show that HBT activates a network of brain regions including the insula, primary somatosensory cortex, and the anterior cingulate cortex, which are regions considered fundamental for both the representation of one’s internal state and for the conscious experience of emotions (Craig, 2002; Critchley et al., 2004; Pollatos et al., 2007, Pollatos et al., 2007).

As regards possible other limitations of our study, we also note that recently, a preprint has appeared among the scientific community, questioning the body perception index as a specific measure of interoceptive sensibility (Gabriele et al., 2020). In this research, which refers to Murphy’s model of interoception (Murphy et al., 2019), the BPQ is shown to be potentially prone to misinterpretation as it seems to confound interoceptive accuracy and interoceptive attention. While we find these insights beneficial for future research, the present study was designed before our awareness of the new model of interoception by (Murphy et al., 2019), and thus we cannot interpret the results of the current study in light of this framework.

Finally, we note that our sample includes, on average, young participants with a rather homogeneous background and that this study is largely based on self-report instruments. While such results may vary on a more general or pathological population, there are multiple occurrences in the literature of alexithymia studies with similar samples (Herbert et al., 2011; Longarzo et al., 2015; Maier et al., 2016; Muir et al., 2017; Scarpazza et al., 2015). Regarding the use of self-report, these instruments imply that individuals with a high level of alexithymia are aware of their problems, which is not always true. Despite this limitation, the TAS-20 is currently the gold standard for the non-clinical assessment of alexithymia.

Overall, future research is thus needed to replicate our findings, using the most recent and innovative ways to measure interoceptive components and heart signals (Murphy, et al., 2018; Owens et al., 2018). Nonetheless, the value of our current results lies in their theoretical and practical implications and in their potential to guide future research.

## Conclusions

In conclusion, we believe that this work provides opening, convincing evidence on the dissociation of three interoceptive components in alexithymia as a candidate mechanism to explain the impaired processing of emotional experience. In this sample and with the current task, individuals with high alexithymic level manifest higher ISb and IAcc, but lower IAw; they also tend to underestimate their interoceptive abilities, while showing no actual interoceptive deficit. Finally, given the significant implications that our findings put forward both for theoretical alexithymia models and practical implications, we call for future research to replicate these results with more recent ways to assess interoceptive components.

## Supplementary Information

Below is the link to the electronic supplementary material.

Supplementary file1 (DOCX 82 KB)

## Data Availability

Data are available upon request.

## Code availability

All analyses were performed in R (R Core Team, 2013).
