# Supplementary material for: Disentangling interoceptive abilities in alexithymia
Source: Psychol Res. 2021 Jun 7;86(3):844–57. doi: 10.1007/s00426-021-01538-x (PMC8182733; doi:10.1007/s00426-021-01538-x)
Supplement: Supplementary file 1 — Supplementary file1 (DOCX 82 KB) [file 426_2021_1538_MOESM1_ESM.docx]

**SUPPLEMENTARY MATERIAL**

**Disentangling Interoception Abilities in Alexithymia**

**Studies investigating the link between alexithymia and interoception.**

| *Interoceptive component* | *Author(s)* | *Year* | *Interoceptive measure(s)* | *Alexithymia measure(s)* | *Results* |
| --- | --- | --- | --- | --- | --- |
| **ISb** | (Zamariola, Maurage, Luminet, & Corneille, 2018) | 2018 | MAIA | TAS-20 | Decreased |
|  | (Muir, Madill, & Brown, 2017) | 2017 | MAIA | TAS-20 | Decreased |
|  | (Brewer, Cook, & Bird, 2016) | 2016 | ICQ | TAS-20 | Decreased |
|  | (Scarpazza, Ladavas, & di Pellegrino, 2015) | 2015 | BPQ | TAS-20; DCPR | Increased |
|  | **(**Longarzo et al., 2015) | 2015 | SAQ | TAS-20 | Increased |
|  | (Ernst et al., 2014) | 2014 | BPQ | TAS-20 | Increased |
| **ISt** | (Nicholson et al., 2018) | 2018 | HPT | TAS-20 | No results |
|  | (Zamariola et al., 2018) | 2018 | HPT | TAS-20 | No results |
|  | (Murphy, Catmur, & Bird, 2018) | 2018 | Muscular effort; taste | TAS-20 | Decreased |
|  | (Murphy, Brewer, Hobson, Catmur, & Bird, 2018) | 2018 | HPT | TAS-20 | No results |
|  | (Bornemann & Singer, 2017) | 2017 | HPT | TAS-20 | No results |
|  | (Scarpazza, Sellitto, & di Pellegrino, 2017) | 2017 | HPT | TAS-20; DCPR | Increased |
|  | (Shah, Hall, Catmur, & Bird, 2016)* | 2016 | HPT | TAS-20 | Decreased |
|  | (Shah, Catmur, & Bird, 2016) | 2016 | HPT | TAS-20 | Decreased |
|  | (Scarpazza et al., 2015) | 2015 | HPT | TAS-20; DCPR | Increased |
|  | (Herbert, Herbert, & Pollatos, 2011) | 2011 | HPT | TAS-20 | Decreased |
|  |  |  |  |  |  |

*Notes*. Ist = Interoceptive Sensitivity; Isb = Interoceptive Sensibility; MAIA = Multimodal Assessment of Interoceptive Awareness Scale; HPT = Heartbeat Perception Task; ICQ = Interoceptive Questionnaire Confusion; BPQ = Body Perception Questionnaire; SAQ = Self Awareness Questionnaire; TAS-20 = 20-item Toronto Alexithymia Scale; DCPR = Diagnostic Criteria for Psychosomatic Research. ^*^Only the first study is included here; the second one involved individuals with autistic spectrum disorders.

**References**.

Bornemann, B., & Singer, T. (2017). Taking time to feel our body: Steady increases in heartbeat perception accuracy and decreases in alexithymia over 9 months of contemplative mental training. *Psychophysiology, 54*(3), 469-482. doi:10.1111/psyp.12790

Brewer, R., Cook, R., & Bird, G. (2016). Alexithymia: a general deficit of interoception. *R Soc Open Sci, 3*(10), 150664. doi:10.1098/rsos.150664

Ernst, J., Boker, H., Hattenschwiler, J., Schupbach, D., Northoff, G., Seifritz, E., & Grimm, S. (2014). The association of interoceptive awareness and alexithymia with neurotransmitter concentrations in insula and anterior cingulate. *Soc Cogn Affect Neurosci, 9*(6), 857-863. doi:10.1093/scan/nst058

Herbert, B. M., Herbert, C., & Pollatos, O. (2011). On the relationship between interoceptive awareness and alexithymia: is interoceptive awareness related to emotional awareness? *J Pers, 79*(5), 1149-1175. doi:10.1111/j.1467-6494.2011.00717.x

Longarzo, M., D'Olimpio, F., Chiavazzo, A., Santangelo, G., Trojano, L., & Grossi, D. (2015). The relationships between interoception and alexithymic trait. The Self-Awareness Questionnaire in healthy subjects. *Front Psychol, 6*, 1149. doi:10.3389/fpsyg.2015.01149

Muir, K., Madill, A., & Brown, C. (2017). Individual differences in emotional processing and autobiographical memory: interoceptive awareness and alexithymia in the fading affect bias. *Cogn Emot, 31*(7), 1392-1404. doi:10.1080/02699931.2016.1225005

Murphy, J., Brewer, R., Hobson, H., Catmur, C., & Bird, G. (2018). Is alexithymia characterised by impaired interoception? Further evidence, the importance of control variables, and the problems with the Heartbeat Counting Task. *Biol Psychol, 136*, 189-197. doi:10.1016/j.biopsycho.2018.05.010

Murphy, J., Catmur, C., & Bird, G. (2018). Alexithymia is associated with a multidomain, multidimensional failure of interoception: Evidence from novel tests. *J Exp Psychol Gen, 147*(3), 398-408. doi:10.1037/xge0000366

Nicholson, T. M., Williams, D. M., Grainger, C., Christensen, J. F., Calvo-Merino, B., & Gaigg, S. B. (2018). Interoceptive impairments do not lie at the heart of autism or alexithymia. *J Abnorm Psychol, 127*(6), 612-622. doi:10.1037/abn0000370

Scarpazza, C., Ladavas, E., & di Pellegrino, G. (2015). Dissociation between Emotional Remapping of Fear and Disgust in Alexithymia. *PLoS One, 10*(10), e0140229. doi:10.1371/journal.pone.0140229

Scarpazza, C., Sellitto, M., & di Pellegrino, G. (2017). Now or not-now? The influence of alexithymia on intertemporal decision-making. *Brain Cogn, 114*, 20-28. doi:10.1016/j.bandc.2017.03.001

Shah, P., Catmur, C., & Bird, G. (2016). Emotional decision-making in autism spectrum disorder: the roles of interoception and alexithymia. *Mol Autism, 7*, 43. doi:10.1186/s13229-016-0104-x

Shah, P., Hall, R., Catmur, C., & Bird, G. (2016). Alexithymia, not autism, is associated with impaired interoception. *Cortex, 81*, 215-220. doi:10.1016/j.cortex.2016.03.021

Zamariola, G., Maurage, P., Luminet, O., & Corneille, O. (2018). Interoceptive accuracy scores from the heartbeat counting task are problematic: Evidence from simple bivariate correlations. *Biol Psychol, 137*, 12-17. doi:10.1016/j.biopsycho.2018.06.006
